# Epigenomics and transcriptomics analyses of multiple system atrophy brain tissue supports a role for inflammatory processes in disease pathogenesis

**DOI:** 10.1186/s40478-020-00946-1

**Published:** 2020-05-14

**Authors:** Conceição Bettencourt, Ignazio S. Piras, Sandrine C. Foti, Joshua Talboom, Yasuo Miki, Tammaryn Lashley, Robert Balazs, Emmanuelle Viré, Thomas T. Warner, Matt J. Huentelman, Janice L. Holton

**Affiliations:** 1 Queen Square Brain Bank for Neurological Disorders, UCL Queen Square Institute of Neurology, London, UK; 2 Department of Clinical and Movement Neurosciences, UCL Queen Square Institute of Neurology, Queen Square Brain Bank for Neurological Disorders, 1 Wakefield street, London, WC1N 1PJ UK; 3 Neurogenomics Division, Translational Genomics Research Institute, Phoenix, AZ USA; 4 Department of Neurodegenerative Disease, UCL Queen Square Institute of Neurology, London, UK; 5 Department of Neuropathology, Institute of Brain Science, Hirosaki University Graduate School of Medicine, Hirosaki, Japan; 6 Institute of Prion Diseases, MRC Prion Unit at UCL, Courtauld Building, 33 Cleveland Street, London, UK; 7 Reta Lila Weston Institute of Neurological Studies, UCL Queen Square Institute of Neurology, London, UK

**Keywords:** Methylomics, DNA methylation, EWAS, Transcriptomics, Gene expression, RNAseq, MSA, Neurodegeneration, Inflammation

To the Editors,

Multiple system atrophy (MSA) is a fatal neurodegenerative disease and its aetiology remains elusive. The pathological hallmark of MSA is the presence of glial cytoplasmic inclusions (GCIs) containing fibrillar α-synuclein in oligodendrocytes [3, 6], but the regional vulnerability of the brain to these GCIs remains poorly understood. We read with interest the paper by Rydbirk and colleagues recently published in *Acta Neuropathologica Communications* [5]. They investigated DNA methylation (5mC) and hydroxymethylation (5hmC) changes in prefrontal cortex samples from MSA patients. We reported previously [1] total DNA methylation (5mC + 5hmC) changes in MSA, and this work by Rydbirk et al. [5] further supports a contribution of epigenetic factors, namely DNA methylation, to MSA brain pathophysiology.

Given the differentially methylated CpGs (e.g. in *AREL1* and *KTN1*), regions (DMRs) and blocks reported for the 5mC fraction by Rydbirk and colleagues [5], we performed additional loci-specific analysis of our MSA DNA methylation data [1]. We used data from our discovery cohort [1], which was composed of neuropathologically confirmed MSA mixed cases and controls, and investigated multiple brain regions characterized by different degrees of GCI burden in MSA, including the cerebellum, and the frontal and occipital lobes. In the frontal lobe, no changes were detected in *AREL1* nor in the reported intergenic CpGs (Supplementary Table S1.1). Although with small effects (absolute delta betas < 5%), two CpGs in *KTN1* were nominally significant (cg14002714 and cg21059882; *p* < 0.05). Regarding the block covering *PHF3*, two CpGs were nominally significant (cg16049132 and cg10435600; *p* < 0.05). Additionally, 52 CpGs in the DMR genes were nominally significant in our data, 14 of which (in 5 genes: *FUT4*, *BCAR1*, *CTSZ*, *ZIC4* and *FERMT3*) demonstrated absolute delta betas of ≥5%. With the exception of cg18023065 in *FUT4*, none of these changes passed multiple testing correction (*p* < 9.07 × 10^− 5^ [0.05/551 CpGs]). Some of those CpGs and additional CpGs were also nominally significant in the other brain regions analysed (Supplementary Table S1.1). Interestingly, the DMR in the *FUT4/PIWIL4* promoter (chromosome 11: 94278407–94,279,068), replicates a DMR we found in the frontal lobe and cross-region analyses of our previous study (Supplementary Tables S3.1 and S3.3 from [1]).

The study by Rydbirk et al. [5] and ours [1] have markedly different designs: a) Rydbirk et al. [5] included white and grey matter from the frontal lobe, whilst we carefully dissected white matter to enrich for oligodendrocytes and analysed different brain regions; and b) they investigated the contributions of 5mC and 5hmC separately. We are aware that in our data alterations in the 5mC and 5hmC proportions can counteract each other and mask the detection of significant changes in total methylation. As an example, the *AREL1* shift from 5mC to 5hmC reported by Rydbirk et al. [5] is masked in our total DNA methylation data, highlighting an advantage of analysing 5mC and 5hmC separately. In addition, distinct cell type compositions in the brain tissue samples may contribute to discordant findings. According to RNAseq data from major brain cell types (data from Zhang et al. [7], Supplementary Fig. 1), our results support methylation changes in genes that are highly expressed in oligodendrocytes, including *KTN1* and *PHF3*, or more highly expressed in microglia/macrophages, including *FUT4*, *CTSZ*, and *FERMT3* (Supplementary Fig. 1). Conversely, genes more highly expressed in neurons and/or with low expression in oligodendrocytes, such as *AREL1*, were less susceptible to DNA methylation changes in our dataset.

Findings from Rydbirk et al. [5] also report increased *AREL1* and MHC class I HLA gene expression in MSA brains. We therefore investigated in our MSA cerebellar white matter RNAseq data [4] gene expression changes in all of the genes reported by Rydbirk et al. [5]. Our study includes two independent cohorts of 66 MSA and 66 healthy controls and laser captured oligodendrocytes [4]. Although we did not find differential expression for *AREL1* or *PHF3*, we found a nominally significant (*p* < 0.05) downregulation of *KTN1* (log2 FC = − 0.465), and upregulation of *CTSZ* (log2 FC = 0.817), *NCS1* (log2 FC = 0.813) and *ZIC4* (log2 FC = 1.520) in some groups of our cohort 1 (Supplementary Table S2). The upregulation of *ZIC4* was replicated in cohort 2 of our study (log2 FC = 1.551) and remained significant when accounting for multiple testing adjustments in the combined analysis of both cohorts 1 and 2 (log2 FC = 1.536; adjusted-*p* = 0.022). In our RNAseq data, MHC class I HLA genes have shown inconsistent results across cohorts/groups, with only *HLA-A* showing nominally significant upregulation in one group of cohort 1 (log2 FC = 1.156 in MSA-P; *p* = 0.002) and *HLA-F* in oligodendrocytes (log2 FC = 1.982; *p* = 0.032). Some of the MHC class I HLA genes, including *HLA-A*, have also shown nominally significant DNA methylation changes in our data (Supplementary Table S1.2).

Overall, we consider that these recent studies by Bettencourt et al. [1], Piras et al. [4], and Rydbirk et al. [5] are complementary, and bring important insights into the brain pathophysiology of MSA. All show changes in DNA methylation or in gene expression levels of genes that are more highly expressed in microglia/macrophages, therefore supporting previous studies highlighting the involvement of inflammatory processes in MSA (e.g. [2]).

## Supplementary information

**Additional file 1: Supplementary Table S1.1.** Loci-specific analysis of differentially methylated CpGs, regions and blocks identified by Rydbirk et al. 2020. **Supplementary Table S1.2.** Loci-specific analysis of differential methylation in MHC class I HLA genes.

**Additional file 2: Supplementary Table S2.** Loci-specific analysis of cerebellar white matter gene expression for genes reported to be differentially methylated and/or differentially expressed by Rydbirk et al. 2020.

**Additional file 3: Supplementary Figure 1.** Boxplots of normalized counts in different brain cell types [7] for the most relevant genes detected in [1, 5]. Raw data were downloaded from Sequencing Reads Archive (*#SRP064454*), pseudoalignment was conducted with *Kallisto v0.46.1*, and counts were normalized with *DESeq2 v1.26.0.* Data were from hippocampus, temporal lobe and fetal cortex*.* Tumor samples were excluded from the dataset. FA: fetal astrocytes. A: astrocytes. N: neurons. O: oligodendrocytes. M: microglia. E: endothelial cells.

## Data Availability

Data generated during this study are included in this published article and its supplementary information files. The datasets used for the analyses during the current study available from the corresponding author on reasonable request.

## Abbreviations

DMRs: Differentially methylated regions
FC: Fold change
GCIs: Glial cytoplasmic inclusions
MSA: Multiple system atrophy
MSA-C: Clinical subtype of multiple system atrophy with a predominant cerebellar phenotype
MSA-P: Clinical subtype of multiple system atrophy with a predominant parkinsonian phenotype
RNAseq: Whole-transcriptome data obtained by next-generation sequencing

## References

[1] Bettencourt C, Foti SC, Miki Y, Botia J, Chatterjee A, Warner TT, Revesz T, Lashley T, Balazs R, Vire E et al (2020) White matter DNA methylation profiling reveals deregulation of HIP1, LMAN2, MOBP, and other loci in multiple system atrophy. Acta Neuropathol 139:135–156. 10.1007/s00401-019-02074-0 31535203 10.1007/s00401-019-02074-0 PMC6942018

[2] Kiely AP, Murray CE, Foti SC, Benson BC, Courtney R, Strand C, Lashley T, Holton JL (2018) Immunohistochemical and molecular investigations show alteration in the inflammatory profile of multiple system atrophy brain. J Neuropathol Exp Neurol 77:598–607. 10.1093/jnen/nly035 29850876 10.1093/jnen/nly035 PMC6005028

[3] Papp MI, Kahn JE, Lantos PL (1989) Glial cytoplasmic inclusions in the CNS of patients with multiple system atrophy (striatonigral degeneration, olivopontocerebellar atrophy and shy-Drager syndrome). J Neurol Sci 94:79–100 2559165 10.1016/0022-510x(89)90219-0

[4] Piras IS, Bleul C, Schrauwen I, Talboom J, Llaci L, De Both MD, Naymik MA, Halliday G, Bettencourt C, Holton JL et al (2020) Transcriptional profiling of multiple system atrophy cerebellar tissue highlights differences between the parkinsonian and cerebellar sub-types of the disease. bioRxiv https://www.biorxiv.org/content/10.1101/2020.02.11.944306v1. Accessed 12 Feb 2020. 10.1186/s40478-020-00950-5 PMC7268362 32493431

[5] Rydbirk R, Folke J, Busato F, Roche E, Chauhan AS, Lokkegaard A, Hejl AM, Bode M, Blaabjerg M, Moller M et al (2020) Epigenetic modulation of AREL1 and increased HLA expression in brains of multiple system atrophy patients. Acta Neuropathol Commun 8:29. 10.1186/s40478-020-00908-7 32151281 10.1186/s40478-020-00908-7 PMC7063795

[6] Spillantini MG, Crowther RA, Jakes R, Cairns NJ, Lantos PL, Goedert M (1998) Filamentous alpha-synuclein inclusions link multiple system atrophy with Parkinson's disease and dementia with Lewy bodies. Neurosci Lett 251:205–208 9726379 10.1016/s0304-3940(98)00504-7

[7] Zhang Y, Sloan SA, Clarke LE, Caneda C, Plaza CA, Blumenthal PD, Vogel H, Steinberg GK, Edwards MS, Li G et al (2016) Purification and characterization of progenitor and mature human astrocytes reveals transcriptional and functional differences with mouse. Neuron 89:37–53. 10.1016/j.neuron.2015.11.013 26687838 10.1016/j.neuron.2015.11.013 PMC4707064

