# Supplementary material for: Epigenomics and transcriptomics analyses of multiple system atrophy brain tissue supports a role for inflammatory processes in disease pathogenesis
Source: Acta Neuropathol Commun. 2020 May 14;8:71. doi: 10.1186/s40478-020-00946-1 (PMC7227350; doi:10.1186/s40478-020-00946-1)
Supplement: Supplementary file 3 — Additional file 3: Supplementary Figure 1. Boxplots of normalized counts in different brain cell types [7] for the most relevant genes detected in [1, 5]. Raw data were downloaded from Sequencing Reads Archive (#SRP064454), pseudoalignment was conducted with Kallisto v0.46.1, and counts were normalized with DESeq2 v1.26.0. Data were from hippocampus, temporal lobe and fetal cortex. Tumor samples were excluded from the dataset. FA: fetal astrocytes. A: astrocytes. N: neurons. O: oligodendrocytes. M: microglia. E: endothelial cells. [file 40478_2020_946_MOESM3_ESM.pdf]

Supplementary Figure 1.

Boxplots of normalized counts in different brain cell types [7] for the most relevant genes detected in [1] and [5]. Raw data were downloaded from Sequencing Reads Archive (*#SRP064454*), pseudoalignment was conducted with *Kallisto v0.46.1*, and counts were normalized with *DESeq2 v1.26.0.* Data were from hippocampus, temporal lobe and fetal cortex. Tumor samples were excluded from the dataset.
FA: fetal astrocytes
A: astrocytes
N: neurons
O: oligodendrocytes
M: microglia
E: endothelial cells

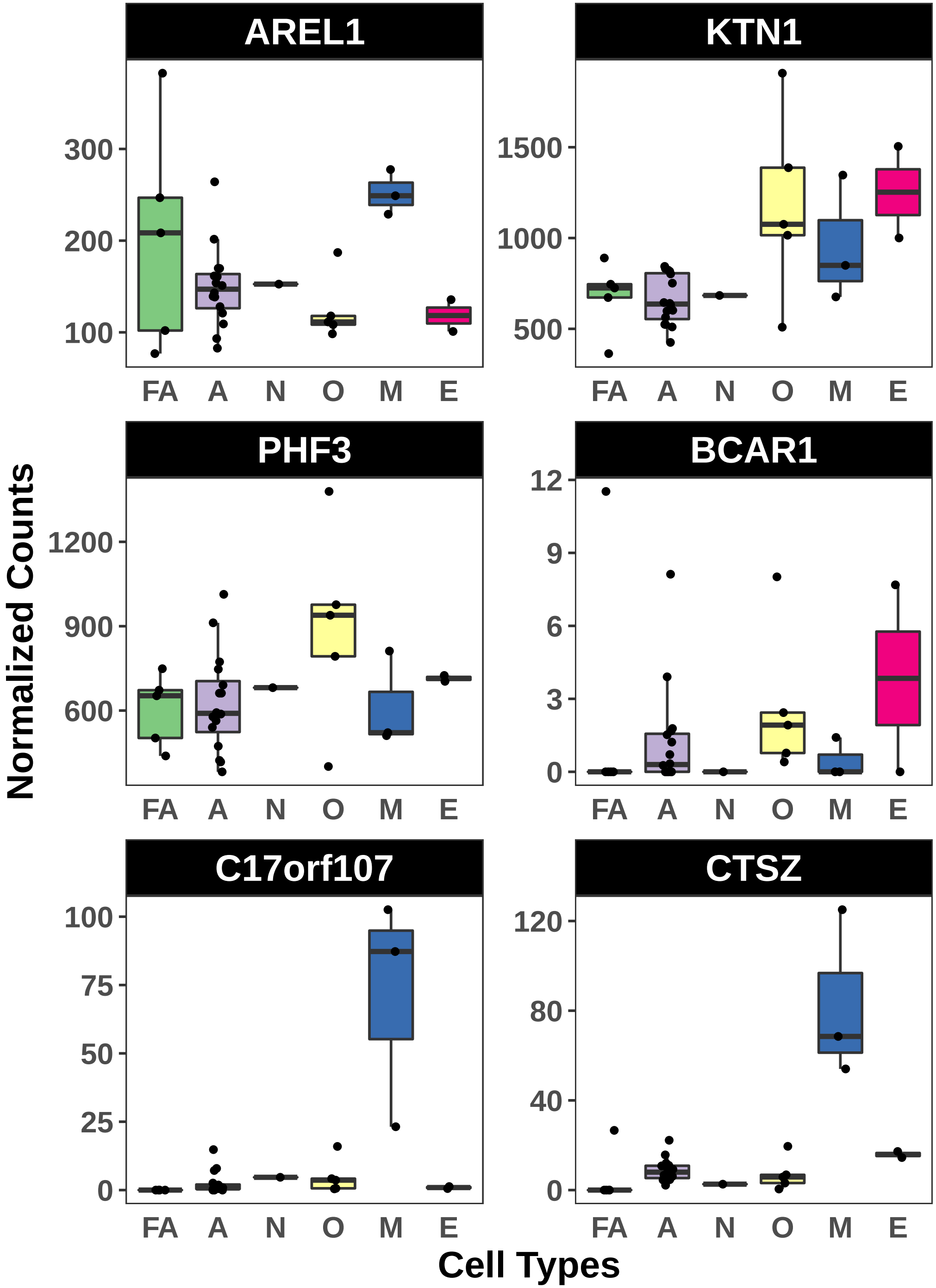
AREL1
KTN1
PHF3
BCAR1
C17orf107
CTSZ
FA
A
N
O
M
E
Normalized Counts
Cell Types
300
200
100
1500
1000
500
1200
900
600
12
9
6
3
0
100
75
50
25
120
80
40

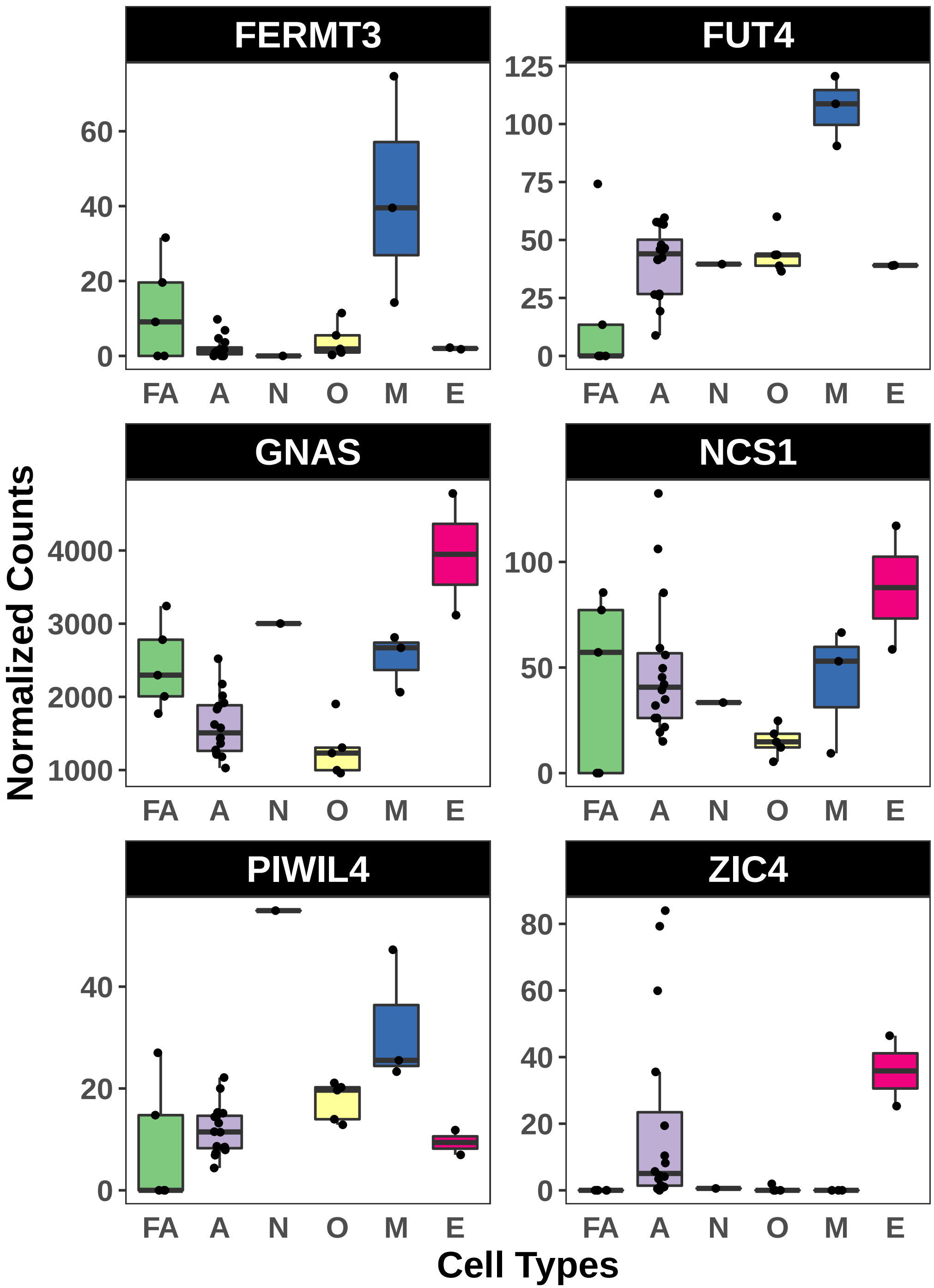
FERMT3
FUT4
GNAS
NCS1
PIWIL4
ZIC4
FA
A
N
O
M
E
Normalized Counts
Cell Types
0
20
40
60
25
50
75
100
125
1000
2000
3000
4000
80

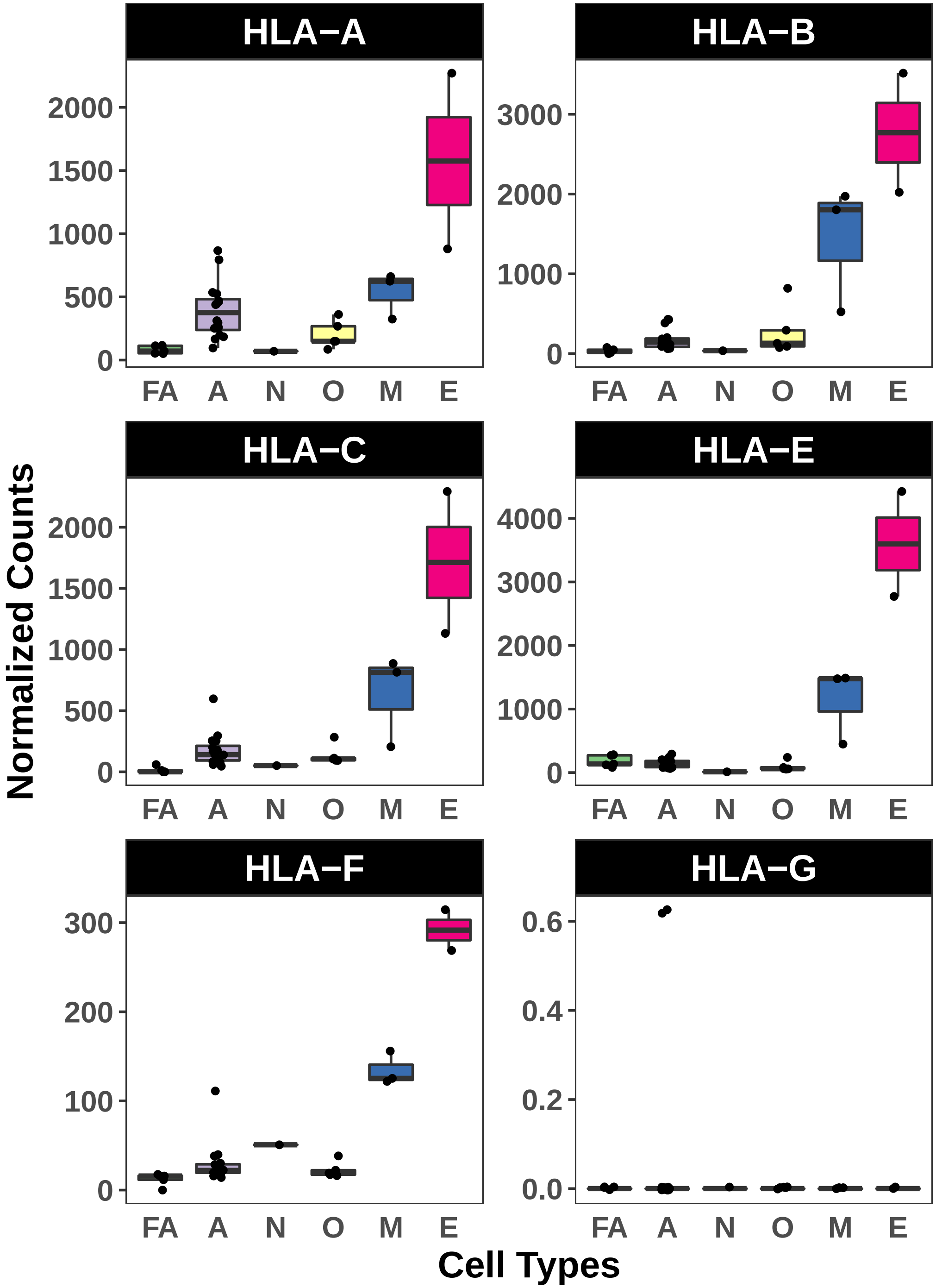
HLA−A
HLA−B
HLA−C
HLA−E
HLA−F
HLA−G
Normalized Counts
Cell Types
FA
A
N
O
M
E
0
500
1000
1500
2000
3000
4000
100
200
300
0.0
0.2
0.4
0.6
